# Supplementary material for: Change in Physical Activity, Sleep Quality, and Psychosocial Variables during COVID-19 Lockdown: Evidence from the Lothian Birth Cohort 1936
Source: Int J Environ Res Public Health. 2020 Dec 30;18(1):210. doi: 10.3390/ijerph18010210 (PMC7795040; doi:10.3390/ijerph18010210)
Supplement: Supplementary file 1 [file ijerph-18-00210-s001.pdf]

International Journal of
Environmental Research and Public Health

MDPI

Supplementary File

Supplementary Table S1

Comparison of participants included and excluded from the analytical sample on covariate variables

	Excluded (N=294)	Included (N=137)	Total (N=431)	*p*
Sex				0.345[1]
- female	156 (53.1%)	66 (48.2%)	222 (51.5%)	
Age 11 IQ				< 0.001[2]
- N-Miss	26	0	26	
- Mean (SD)	100.566 (15.532)	105.874 (13.239)	102.361 (14.992)	
Years of education				0.002[2]
- Mean (SD)	10.776 (1.155)	11.153 (1.175)	10.896 (1.174)	
Occupational class				< 0.001[1]
- N-Miss	7	0	7	
- professional	51 (17.8%)	49 (35.8%)	100 (23.6%)	
- managerial	114 (39.7%)	54 (39.4%)	168 (39.6%)	
- skilled non-manual	73 (25.4%)	19 (13.9%)	92 (21.7%)	
- skilled manual	39 (13.6%)	12 (8.8%)	51 (12.0%)	
- partly skilled	8 (2.8%)	3 (2.2%)	11 (2.6%)	
- unskilled	2 (0.7%)	0 (0.0%)	2 (0.5%)	
History of diabetes				0.479[1]
- yes	37 (12.6%)	14 (10.2%)	51 (11.8%)	
History of CVD				0.319[1]
- N-Miss	2	0	2	
- yes	111 (38.0%)	59 (43.1%)	170 (39.6%)	

HADS anxiety				0.057[2]
- N-Miss	3	0	3	
- Mean (SD)	4.347 (2.976)	3.759 (2.949)	4.159 (2.977)	
HADS depression				0.140[2]
- N-Miss	3	0	3	
- Mean (SD)	3.244 (2.469)	2.876 (2.257)	3.126 (2.407)	
Extraversion				0.250[2]
- N-Miss	25	0	25	
- Mean (SD)	21.216 (7.058)	22.095 (7.678)	21.512 (7.275)	
Agreeableness				0.984[2]
- N-Miss	20	0	20	
- Mean (SD)	30.887 (5.196)	30.898 (5.447)	30.891 (5.274)	
Conscientiousness				0.133[2]
- N-Miss	22	0	22	
- Mean (SD)	27.184 (6.089)	28.131 (5.862)	27.501 (6.023)	
Emotional stability				0.031[2]
- N-Miss	23	0	23	
- Mean (SD)	25.177 (6.665)	26.737 (7.255)	25.701 (6.900)	
Intellect				0.020[2]
- N-Miss	23	0	23	
- Mean (SD)	23.129 (5.912)	24.562 (5.789)	23.610 (5.903)	
Lives alone*				0.381[1]
- N-Miss	253	0	253	
- yes	19 (46.3%)	53 (38.7%)	72 (40.4%)	

Note. *Assessed as part of the LBC1936 COVID questionnaire,[1] Pearson's Chi-squared test, [2] Linear Model ANOVA.

Supplementary Table S2

Comparison of participants included and excluded from the analytical sample on cognitive test scores at age 82

	Excluded (N=294)	Included (N=137)	Total (N=431)	*p*
Digit span backward				0.010[1]
- N-Miss	3	2	5	
- Mean (SD)	6.993 (2.256)	7.615 (2.437)	7.190 (2.330)	
Symbol search				< 0.001[1]
- N-Miss	14	2	16	
- Mean (SD)	21.257 (6.999)	24.193 (6.365)	22.212 (6.930)	
Digit symbol				< 0.001[1]
- N-Miss	10	3	13	
- Mean (SD)	49.384 (13.074)	54.366 (11.497)	50.981 (12.790)	
Matrix reasoning				< 0.001[1]
- N-Miss	9	4	13	
- Mean (SD)	12.137 (5.055)	14.617 (5.184)	12.926 (5.220)	
Letter number sequencing				< 0.001[1]
- N-Miss	9	2	11	
- Mean (SD)	9.095 (2.939)	10.230 (2.440)	9.460 (2.835)	
Block design				< 0.001[1]
- N-Miss	7	4	11	
- Mean (SD)	28.362 (9.279)	33.248 (9.449)	29.910 (9.595)	

Note. [1] Linear Model ANOVA

Supplementary Table S3

Comparison of participants included and excluded from the analytical sample on outcome variables assessed at baseline

	Excluded (N=294)	Included (N=137)	Total (N=431)	*p*
Physical activity				0.242[1]
- N-Miss	19	0	19	
- only household chores	44 (16.0%)	14 (10.2%)	58 (14.1%)	
- outdoor activities 1-2 x per week	56 (20.4%)	28 (20.4%)	84 (20.4%)	
- outdoor activities >2 x per week	138 (50.2%)	67 (48.9%)	205 (49.8%)	
- moderate exercise 1-2 x per week	20 (7.3%)	19 (13.9%)	39 (9.5%)	
- moderate exercise >2 x per week	13 (4.7%)	6 (4.4%)	19 (4.6%)	
- keep-fit/heavy exercise several x per week	4 (1.5%)	3 (2.2%)	7 (1.7%)	
Sleep quality				0.404[1]
- N-Miss	16	0	16	
- very bad	9 (3.2%)	1 (0.7%)	10 (2.4%)	
- fairly bad	38 (13.7%)	16 (11.7%)	54 (13.0%)	
- fairly good	152 (54.7%)	80 (58.4%)	232 (55.9%)	
- very good	79 (28.4%)	40 (29.2%)	119 (28.7%)	
Loneliness				0.235[1]
- N-Miss	16	0	16	
- none/almost none of the time	224 (80.6%)	111 (81.0%)	335 (80.7%)	
- some of the time	50 (18.0%)	22 (16.1%)	72 (17.3%)	
- most of the time	2 (0.7%)	4 (2.9%)	6 (1.4%)	

Note. [1] Pearson's Chi-squared test, [2] Linear Model ANOVA.

- all or almost all the time	2 (0.7%)	0 (0.0%)	2 (0.5%)	
Memory problems				0.046[2]
- N-Miss	20	0	20	
- Mean (SD)	1.449 (1.176)	1.212 (1.046)	1.370 (1.139)	
Wellbeing				0.787[2]
- N-Miss	19	0	19	
- Mean (SD)	37.218 (8.231)	37.453 (8.369)	37.296 (8.268)	
Social support				0.668[2]
- N-Miss	12	0	12	
- Mean (SD)	12.858 (2.065)	12.759 (2.490)	12.826 (2.210)	
Neighbourhood cohesion				0.488[2]
- N-Miss	13	0	13	
- Mean (SD)	22.530 (5.392)	22.920 (5.377)	22.658 (5.384)	

Supplementary Table S4

Correlation between outcome variables at baseline (T1)

	1	2	3	4	5	6
1. Sleep T1						
2. Physical activity T1	0.154					
3. Loneliness T1	-0.189*	-0.190*				
4. Memory problems T1	-0.117	-0.053	0.161			
5. Wellbeing T1	0.293**	0.230**	-0.478**	-0.406**		
6. Social support T1	0.242**	0.151	-0.330**	-0.171*	0.428**	
7. Neighborhood T1	0.015	0.068	-0.165	0.030	0.131	0.239**

Note. Correlations are Spearman's rho. * $p<0.05$, ** $p<0.01$

Supplementary Table S5

Correlation between outcome variables during lockdown (T2)

	1	2	3	4	5	6
1. Sleep T2						
2. Physical activity T2	0.144					
3. Loneliness T2	-0.251**	-0.018				
4. Memory problems T2	0.062	0.043	0.053			
5. Wellbeing T2	0.175*	0.133	-0.389**	-0.347**		
6. Social support T2	0.070	0.189*	-0.147	-0.182*	0.487**	
7. Neighborhood T2	0.168	0.243**	-0.147	0.041	0.222**	0.367**

Note. Correlations are Spearman's rho. * $p<0.05$, ** $p<0.01$

Supplementary Table S6

Results from univariate analysis predicting change in wellbeing

Variable	*β*	95% CI	*p*	FDR *p*
Education	-0.147	-0.301,0.007	0.061	0.259
Anxiety	-0.120	-0.272,0.033	0.124	0.355
Depression	-0.122	-0.295,0.051	0.167	0.355
Intellect	-0.047	-0.244,0.150	0.639	0.821
Conscientiousness	0.012	-0.140,0.163	0.878	0.878
Extraversion	0.035	-0.128,0.197	0.676	0.821
Agreeableness	0.017	-0.169,0.203	0.858	0.878
**Emotional stability***	0.230	0.092,0.369	0.001	0.017
Age 11 IQ	-0.078	-0.223,0.067	0.293	0.553
Fluid g	0.040	-0.107,0.186	0.597	0.821
**Living with others**	0.332	0.014,0.651	0.041	0.232
Occupational class				
managerial-technical	0.247	-0.078,0.573	0.136	0.355
skilled non-manual	0.340	-0.129,0.808	0.156	0.355
manual	0.265	-0.287,0.818	0.347	0.59
Sex	-0.047	-0.363,0.270	0.773	0.876
History of Diabetes	0.108	-0.304,0.520	0.607	0.821
**History of CVD***	-0.421	-0.710,-0.133	0.004	0.034

Note. Bold typeface denotes $p < 0.05$. *Survive correction for multiple comparisons. Estimates from latent change score model additionally adjusting for wellbeing at T1, each covariate variable is entered separately. Estimates are standardized, for binary variables estimates represent a change in the dependent variable in standard deviation units when the binary covariate changes from zero to one. Occupational class is dummy coded with professional as the reference category.

Supplementary Table S7

Results from univariate analysis predicting change in social support

Variable	*β*	95% CI	*p*	FDR *p*
Education	-0.114	-0.232,0.004	0.057	0.193
Anxiety	-0.038	-0.156,0.081	0.533	0.630
Depression	-0.095	-0.232,0.042	0.176	0.332
**Intellect**	0.109	0.004,0.213	0.041	0.193
Conscientiousness	0.046	-0.107,0.200	0.556	0.630
Extraversion	0.130	-0.007,0.267	0.063	0.193
Agreeableness	0.123	-0.030,0.275	0.115	0.244
Emotional stability	0.116	-0.007,0.239	0.065	0.193
Age 11 IQ	-0.060	-0.158,0.038	0.230	0.370
Fluid g	0.082	-0.061,0.224	0.261	0.370
Living with others	0.198	-0.045,0.442	0.111	0.244
Occupational class				
managerial-technical	0.148	-0.109,0.406	0.259	0.370
skilled non-manual	-0.151	-0.531,0.230	0.439	0.574
**manual**	0.539	0.147,0.930	0.007	0.119
Sex	0.022	-0.237,0.281	0.867	0.921
History of Diabetes	-0.003	-0.446,0.440	0.989	0.989
History of CVD	-0.239	-0.494,0.017	0.068	0.193

Note. Bold typeface denotes $p < 0.05$. Estimates from latent change score model additionally adjusting for social support at T1, each covariate variable is entered separately. Estimates are standardized, for binary variables estimates represent a change in the dependent variable in standard deviation units when the binary covariate changes from zero to one. Occupational class is dummy coded with professional as the reference category.

Supplementary Table S8

Results from univariate analysis predicting change in physical activity

Variable	*β*	95% CI	*p*	FDR *p*
Education	0.106	-0.030,0.243	0.126	0.306
Anxiety	-0.021	-0.178,0.136	0.792	0.898
Depression	-0.010	-0.177,0.156	0.902	0.902
**Intellect**	0.135	0.008,0.261	0.037	0.126
Conscientiousness	0.071	-0.055,0.196	0.270	0.519
**Extraversion***	0.185	0.062,0.309	0.003	0.013
Agreeableness	0.034	-0.113,0.181	0.648	0.898
Emotional stability	0.033	-0.112,0.179	0.652	0.898
**Age 11 IQ***	0.212	0.081,0.342	0.002	0.011
**Fluid g***	0.233	0.094,0.372	0.001	0.008
Living with others	-0.255	-0.531,0.020	0.069	0.196
Occupational class				
managerial-technical	-0.164	-0.458,0.130	0.275	0.519
skilled non-manual	0.042	-0.434,0.518	0.863	0.902
**manual***	-0.703	-1.129,-0.277	0.001	0.008
Sex	0.048	-0.230,0.326	0.735	0.898
History of Diabetes	-0.069	-0.564,0.426	0.786	0.898
History of CVD	-0.128	-0.414,0.158	0.380	0.646

Note. Bold typeface denotes $p < 0.05$. *Survives correction for multiple comparisons. Estimates from latent change score model additionally adjusting for physical activity at T1, each covariate variable is entered separately. Estimates are standardized, for binary variables estimates represent a change in the dependent variable in standard deviation units when the binary covariate changes from zero to one. Occupational class is dummy coded with professional as the reference category.

Supplementary Table S9

Results from univariate analysis predicting change in neighbourhood cohesion

Variable	*β*	95% CI	*p*	FDR *p*
Education	-0.027	-0.176,0.123	0.727	0.970
Anxiety	0.053	-0.096,0.201	0.488	0.948
Depression	-0.064	-0.191,0.063	0.321	0.891
**Intellect**	0.157	0.030,0.285	0.015	0.162
Conscientiousness	-0.014	-0.135,0.107	0.817	0.970
Extraversion	0.041	-0.120,0.202	0.617	0.954
Agreeableness	0.105	-0.038,0.247	0.149	0.633
Emotional stability	0.008	-0.145,0.160	0.923	0.970
Age 11 IQ	0.053	-0.062,0.167	0.367	0.891
Fluid g	0.154	0.002,0.305	0.046	0.261
Living with others	-0.024	-0.322,0.273	0.874	0.970
Occupational class				
managerial-technical	-0.006	-0.334,0.321	0.970	0.970
skilled non-manual	0.013	-0.365,0.391	0.947	0.970
manual	0.169	-0.412,0.750	0.568	0.954
Sex	0.098	-0.188,0.383	0.502	0.948
History of Diabetes	0.241	-0.149,0.631	0.225	0.765
**History of CVD**	-0.328	-0.604,-0.053	0.019	0.162

Note. Bold typeface denotes $p < 0.05$. Estimates from latent change score model additionally adjusting for neighbourhood cohesion at T1, each covariate variable is entered separately. Estimates are standardized, for binary variables estimates represent a change in the dependent variable in standard deviation units when the binary covariate changes from zero to one. Occupational class is dummy coded with professional as the reference category.

Supplementary Table 10

Results from univariate analysis predicting change in memory problems

Variable	*β*	95% CI	*p*	FDR *p*
Education	-0.015	-0.182,0.152	0.860	0.860
Anxiety	0.021	-0.153,0.194	0.815	0.860
**Depression**	-0.159	-0.316,-0.002	0.047	0.493
Intellect	0.022	-0.142,0.186	0.795	0.860
Conscientiousness	-0.061	-0.233,0.111	0.489	0.860
Extraversion	0.015	-0.147,0.177	0.854	0.860
Agreeableness	0.139	-0.005,0.282	0.058	0.493
Emotional stability	-0.037	-0.197,0.123	0.649	0.860
Age 11 IQ	0.071	-0.083,0.225	0.367	0.860
Fluid g	-0.056	-0.217,0.105	0.498	0.860
Living with others	-0.165	-0.486,0.157	0.315	0.860
Occupational class				
managerial-technical	0.074	-0.296,0.444	0.696	0.860
skilled non-manual	0.191	-0.260,0.641	0.408	0.860
manual	0.224	-0.368,0.817	0.458	0.860
Sex	-0.189	-0.512,0.133	0.250	0.860
History of Diabetes	0.042	-0.404,0.488	0.853	0.860
History of CVD	-0.079	-0.413,0.254	0.642	0.860

Note. Bold typeface denotes $p < 0.05$. Estimates from latent change score model additionally adjusting for memory problems at T1, each covariate variable is entered separately. Estimates are standardized, for binary variables estimates represent a change in the dependent variable in standard deviation units when the binary covariate changes from zero to one. Occupational class is dummy coded with professional as the reference category.

Supplementary Table S11

Results from univariate analysis predicting change in loneliness

Variable	OR	95% CI	*p*	FDR *p*
Years of education	0.943	0.679,1.312	0.729	0.923
**Anxiety**	1.187	1.032,1.365	0.016	0.128
Depression	0.952	0.791,1.144	0.597	0.923
Intellect	0.980	0.915,1.049	0.557	0.923
Conscientiousness	1.015	0.951,1.085	0.649	0.923
Extraversion	0.994	0.944,1.046	0.808	0.923
Agreeableness	0.998	0.928,1.073	0.953	0.953
Emotional stability	0.946	0.891,1.004	0.069	0.368
Age 11 IQ	0.987	0.959,1.015	0.361	0.825
Fluid g	0.984	0.659,1.471	0.938	0.953
**Living with others***	0.165	0.070,0.389	<0.001	0.016
Occupational class				
managerial-technical	0.816	0.323,2.064	0.667	0.923
skilled non-manual/manual	1.672	0.627,4.460	0.304	0.811
Sex	1.766	0.805,3.872	0.156	0.624
History of Diabetes	1.185	0.335,4.190	0.792	0.923
History of CVD	1.685	0.763,3.721	0.197	0.630

Note. Bold typeface denotes $p < 0.05$. *Survives correction for multiple comparisons. OR = odds ratio. Estimates are from ordinal logistic regression models predicting change in loneliness (increase, no change, or decrease) and adjusting for loneliness at T1 (none of the time vs some of the time or more). Skilled non-manual and manual occupational classes were collapsed for this analysis.

Supplementary Table S12

Results from univariate analysis predicting change in sleep quality

Variable	OR	95% CI	*p*
Years of education	0.954	0.708,1.286	0.756
Anxiety	1.069	0.947,1.207	0.282
Depression	0.890	0.758,1.044	0.153
Intellect	1.053	0.988,1.121	0.111
Conscientiousness	1.010	0.949,1.075	0.745
Extraversion	0.984	0.939,1.031	0.488
Agreeableness	1.019	0.954,1.089	0.568
Emotional stability	0.984	0.936,1.033	0.512
Age 11 IQ	1.005	0.979,1.032	0.693
Fluid g	1.309	0.915,1.873	0.140
Living with others	0.980	0.472,2.034	0.957
Occupational class			
managerial-technical	0.779	0.339,1.793	0.557
skilled non-manual	0.836	0.275,2.537	0.752
manual	0.762	0.23,2.528	0.657
Sex	1.234	0.607,2.507	0.561
History of Diabetes	1.306	0.392,4.354	0.664
History of CVD	0.617	0.300,1.270	0.190

Note. OR = odds ratio. Results are from univariate ordinal logistic regression models predicting change in sleep quality (increase, no change, or decrease) and adjusting for sleep quality at T1 (very or fairly bad vs very or fairly good).

Supplementary Table S13

Comparison of physical activity and social support levels between Waves 4 and 5 of the LBC1936 study

	Wave 4 (N=137)	Wave 5 (N=137)	*p*
Physical activity			0.129[1]
- only household chores	6 (4.4%)	14 (10.2%)	
- outdoor activities 1-2 x per week	30 (21.9%)	28 (20.4%)	
- outdoor activities >2 x per week	77 (56.2%)	67 (48.9%)	
- moderate exercise 1-2 x per week	10 (7.3%)	19 (13.9%)	
- moderate exercise >2 x per week	10 (7.3%)	6 (4.4%)	
- keep-fit/heavy exercise several x per week	4 (2.9%)	3 (2.2%)	
Social support			0.360[1]
- Mean (SD)	12.927 (1.973)	12.759 (2.490)	
